# Virulence of H5N1 virus in mice attenuates after *in vitro *serial passages

**DOI:** 10.1186/1743-422X-8-93

**Published:** 2011-03-04

**Authors:** Jing Li, Bohua Liu, Guohui Chang, Yi Hu, Dawei Zhan, Yukun Xia, Yongqiang Li, Yinhui Yang, Qingyu Zhu

**Affiliations:** 1 State Key Laboratory of Pathogens and Bio-security, Beijing Institute of Microbiology and Epidemiology, Beijing 100071, China

## Abstract

The virulence of A/Vietnam/1194/2004 (VN1194) in mice attenuated after serial passages in MDCK cells and chicken embryos, because the enriched large-plaque variants of the virus had significantly reduced virulence. In contrast, the small-plaque variants of the virus and the variants isolated from the brain of mice that were infected with the parental virus VN1194 had much higher virulence in mice. The virulence attenuation of serially propagated virus may be caused by the reduced neurotropism in mice. Our whole genome sequence analysis revealed substitutions of a total of two amino acids in PB1, three in PB2, two in PA common for virulence attenuated variants, all or part of which may be correlated with the virulence attenuation and reduced neurotropism of the serially propagated VN1194 in mice. Our study indicates that serial passages of VN1194 *in vitro *lead to adaptation and selection of variants that have markedly decreased virulence and neurotropism, which emphasizes the importance of direct analysis of original or less propagated virus samples.

## Backgrounds

H5N1 avian influenza virus continues to cause human infections with high mortality, posing a serious pandemic threat to human health. Although human-to-human transmission of the virus has not yet been very efficient [1-4] up to now, more transmissible and sustained variants of H5N1 virus may arise in human populations and other mammalian hosts through accumulating mutations [5]. Mounting evidence is showing that some H5N1 strains, including those isolated from humans, are mixed in population [6,7] and composed of heterogeneous variants that can be differentially selected by human or mammalian hosts. Some studies indicated that the virulence of avian influenza virus is altered after the virus' transmission to its mammalian hosts [8,9]. It has also been reported that influenza viruses are affected by the culture systems [10-12]. For instance, viruses with different specific amino acid residues at the same sites of PB2 are selected differentially after their growth adaptation in different culture systems [10]. Furthermore, some specific amino acid changes in HA molecule were observed mainly around the receptor binding site [12].

A/Vietnam/1194/2004 is a clade 1 H5N1 influenza virus strain originally isolated from a fatal human case in 2004. It is also highly pathogenic to mice, which is one of mammalian infection models to determine the virulence and pathogenic mechanism of H5N1 influenza virus [7,13-16]. The virus proliferation and the proliferation-induced pathological immune reactions are the major causes of damages to host tissues and organs. Besides, the invasiveness of the central nervous system also contributes to the high virulence of some H5N1 strains [2,17,18], and highly pathogenic H5N1 viruses are neuro-virulent to birds, mice, and ferrets to cause pathological damages in the central nervous system [19-25].

In this current study, we investigated the differences between the wild A/Vietnam/1194/2004 virus and the serially propagated virus in plaque forming, virulence and genome sequences. We further determined the attenuation of the serially propagated virus in mouse model and its potential mechanisms.

## Materials and methods

### Cells and viruses

Madin-Darby Canine Kidney (MDCK) and A549 (human lung epithelial cell line) cells were cultured in DMEM (Invitrogen, U.S.A) supplemented with 10% FBS (Invitrogen, U.S.A). The wild A/Vietnam/1194/2004 (VN1194-W) virus had been propagated two times (35°C) in 10-day-old SPF chick embryos. The serial-propagated A/Vietnam/1194/2004 virus was developed from VN1194-W after four times of proliferations (35°C) in SPF chick embryos and three times of proliferations in MDCK cells (35°C) (VN1194-P). The mouse lung variant (1194-ML) and the mouse brain variant (1194-MB) were isolated separately from the lungs and brains of mice infected with VN1194-P and then propagated for one time in MDCK cells (35°C). The virus of large-plaque variant (1194-LP) and the small-plaque variant (1194-SP) that were isolated from VN1194-P *in vitro *had been propagated for one time in MDCK cells (35°C). All of the experiments with live H5N1 viruses were done in a bio-safety level three containment laboratory approved by the Biosafety Management Committee of State Key Laboratory of Pathogens and Bio-security.

### Plaque assay and variants selection

Confluent monolayer MDCK cells were inoculated with 10-fold serially diluted H5N1 virus at 35°C for one hour (h). After the removal of the inoculum, cells were washed once with Earle solution and overlaid with 1% hypo-Tm(temperature)-solved agarose containing 0.5% lactalbumin hydrolyzate, 0.5‰ glutamine, and 10% FBS. After two days (d) inoculation at 35°C, cells were stained with 0.25‰ neutral red containing 1% agarose, and the plaque morphology was evaluated. 1194-LP and 1194-SP were serially plaque-purified for three times from the larger plaque and the smaller plaque formed by VN1194-P until homogeneous large or small plaques were formed. Mice were infected with 10^3 ^plaque-forming units (pfu) of VN1194-P intranasally (i.n.) and were euthanized on days three post inoculation (p.i.), then their lungs and brains were collected and titrated separately for virus plaque forming assay and virulence determination.

### Growth Curves of plaque-purified isolates

MDCK and A549 cells were inoculated with H5N1 virus at a 0.01 MOI (multiplicity of infection), respectively. Then they were maintained in DMEM medium containing 2% FBS at 35°C. At time points of eight, 16, 48 and 72 hours, supernatants of A549 cells, and at time points of eight, 24, 36 and 48 hours, supernatants of MDCK cells were collected and determined by the plaque titer on MDCK cells. The growth curves were determined in three independent experiments.

### Virulence of plaque-purified isolates

Six groups of six to 8-week-old, 15-17 g female BALB/c mice (Beijing Experimental Animal Center) were lightly anesthetized with pentobarbital and inoculated i.n. with 50 μl (100 pfu) of VN1194-W, VN1194-P, 1194-SP, 1194-LP, 1194-ML, or 1194-MB. 10 mice in each group were monitored for 14 days and the mean survival time was estimated by the Kaplan-Meier method. Fisher's exact test was used to analyze the differences in survival rates when there were no censored observations present. Three mice in groups of VN1194-W, VN1194-P, 1194-LP were euthanized on days three and six p.i.. Mice brains, bloods and lungs were collected and titrated for virus infectivity by TCID_50_. The 50% mouse lethal dose (MLD_50_) of each virus was determined by inoculating five mice in each group with 10-fold serial dilutions of the virus in a 50 μl volume, and calculated by the method of Reed and Muench [26]. In another experiment, three groups of mice were lightly anesthetized with pentobarbital and inoculated intravenously (i.v.) with 25 μl (50 pfu) of VN1194-W, VN1194-P, or 1194-LP. Three mice in each group were euthanized on days three and six p.i.. Mice brains, bloods and lungs were collected and titrated for virus infectivity by TCID_50_. The remaining 10 mice in each group were monitored for 14 days for mortality and the average survival times of each group, which were also tested by Kaplan-Meier method and Fisher's exact test.

### Sequencing of viruses

VN1194-W, 1194-SP, 1194-LP, 1194-ML and 1194-MB were fully sequenced to find the mutations. Viral RNA was extracted (Qiagen, Maryland, U.S.A) from virus stocks and reverse-transcribed by reverse transcriptase AMV (Promega, Madison, U.S.A). Genes of eight influenza fragments were amplified by PCR with a set of fragment specific primers (Invitrogen, Shanghai, China) and DNA polymerase (PFU polymerase, Promega, Madison, U.S.A). The PCR products were gel-purified with a QIAquick gel extraction kit (Qiagen, Maryland, U.S.A) and cloned into pGEM-T-Easy (Promega, Madison, U.S.A). The nucleotide sequences of the viral genes were analyzed by MEGA 4.

## Results

### Plaque forming property of wild, propagated and plaque-purified isolates in MDCK cells

**Figure 1:**
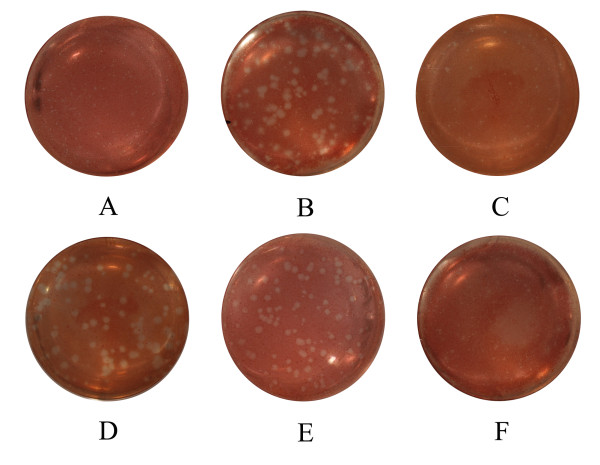
**Plaque forming assay of VN1194-W, VN1194-P and other variants isolated from VN1194**. Confluent monolayer of MDCK cells were inoculated with 10-fold serial dilutions of H5N1 virus and overlaid with 1% hypo-Tm-solved agarose. After two d inoculation at 35°C, cells were stained with neutral red and plaque morphology was evaluated. The larger plaques were discriminated from smaller plaques when their size was two times larger than smaller ones. A: VN1194-W; B: VN1194-P; C: 1194-SP; D: 1194-LP; E: 1194-ML; F: 1194-MB.

The plaques formed by VN1194-W, propagated for only two times in chicken embryo, were homogeneously punctiform in size (Figure 1A). The plaques formed by VN1194-P, however, were heterogeneous in morphology with more than 50% of them being larger than the others (Figure 1B). The heterogeneity of VN1194-P plaques was confirmed by plaque reformation assays of 1194-SP and 1194-LP, plaque-purified smaller and larger variants isolated from VN1194-P (Figure 1C and 1D). And the size of 1194-SP and VN1194-W were seven times less than 1194-LP (Table 1).

**Table 1:** Plaque forming property and virulence of VN1194-W and other viruses

Virus	Passage history*	Titer (pfu/mL)	Plaque size ^#^ (mm)	MLD_50_^‡^	Survival time (i.n.) †	survival time (i.v.) †
VN1194-W	Egg × 2	4.2×10^8^	0.096±0.038	0.66	8.1±0.43	5.6±0.40
VN1194-P	Egg × 4; MDCK ×3	4.88×10^6^	Mixed ND§	1.54×10^3^	14	13.4±0.57
1194-SP	MDCK ×1	1.67×10^6^	0.080±0.026	52.87	7.6±0.71	ND§
1194-LP	MDCK ×1	6.50×10^6^	0.746±0.225	6.50×10^4^	14	14
1194-ML	MDCK ×1	1.85×10^6^	Mixed, ND§	3.95×10^2^	13.3±0.66	ND§
1194-MB	MDCK ×1	4.40×10^7^	0.079±0.034	87.6	10.8±1.00	ND§

* Prepared in chicken embryos or MDCK cells for 48-60 hours.

^# ^Average plaque size(mm, means ± SEs)

^‡ ^Expressed as the pfu to give 1 LD_50_.

† Average survival time (days) (means ± SEs of 10 mice per group) after intranasal or intravenous infection.

Mean survival time was estimated by the Kaplan-Meier method. Fisher's exact test was used to analyze the differences in survival rates between groups when no censored observations presented. Significant differences were observed in mean survival time between VN1194-W group and VN1194-P group and between 1194-LP group and 1194-SP group (P < 0.01), No significant differences were observed between VN1194-W group and 1194-SP group (P > 0.05). Significant differences were observed between VN1194-W group and 1194-MB group (P < 0.05), but no significant differences between 1194-ML group and VN1194-P group (P > 0.05)

§ ND: not determined.

### Growth curves of plaque-purified viruses

**Figure 2:**
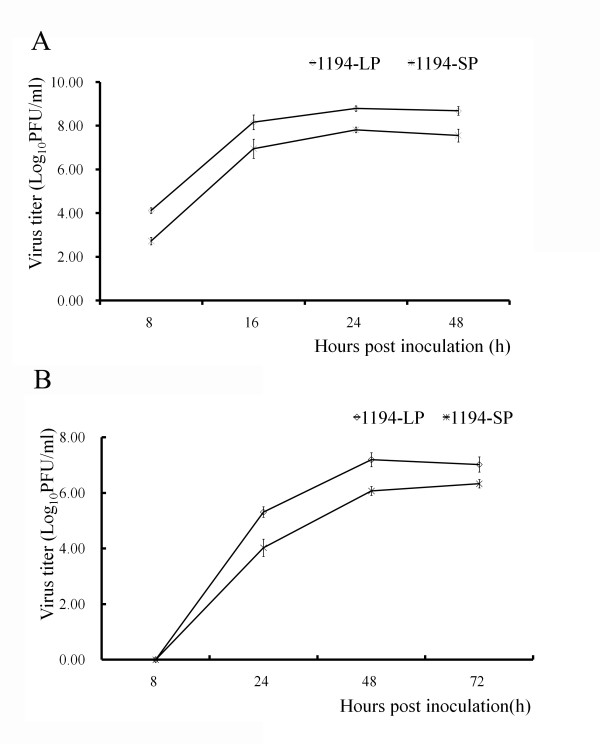
**Growth curves of 1194-LP and 1194-SP**. Cells were inoculated at a 0.01 MOI (multiplicity of infection). The plaque titers of the supertant of MDCK (A) or A549 (B) cells infected with 1194-LP (◊) or 1194-SP (*) were determined at appropriate time points on MDCK cells. Each curve is the average of three independent experiments.

Because the larger plaque variants in VN1194-P developed from VN1194-W after serial *in vitro *passages, the growth of 1194-LP and 1194-SP were determined in MDCK and A549 cells, two commonly used mammalian cells, to confirm whether larger plaques in VN1194-P had been enriched because of higher replication efficiency. Figure 2 shows that both 1194-LP and 1194-SP grew to the highest titer in MDCK cells at 24 h post inoculation and to the highest titer in A549 cells at 48 h post inoculation. And the rate of replication and virus yield of 1194-LP in two kinds of cells were approximately 100 or 10-fold higher than 1194-SP.

### Attenuation of VN1194 in virulence to mice after serial passages in vitro

**Figure 3:**
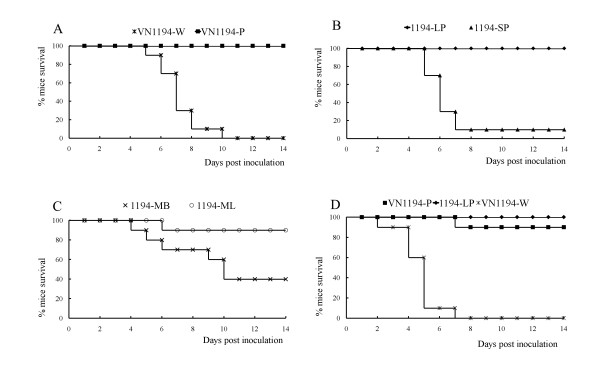
**Survival rates of BALB/c mice infected with VN1194-W, VN1194-P and other variants**. Six to 8-week-old, 15-17 g femal mice with 10 in each group were infected i.n. with 100 pfu (A-C) and i.v. with 50 pfu (D).

The virulence of VN1194 with different passage histories was determined in mice. As shown in Figure 3A, the parental virus (VN1194-W) caused lethal infection in all mice by day 10 p.i. in a dose of 100 pfu. In contrast, all mice infected with serial-propagated virus (VN1194-P) survived until at least day 14 p.i. (*P *< 0.001). This virulence difference was further confirmed by their MLD_50 _and the mouse average survival time (Table 1). To explore the possible mechanism of VN1194-P attenuation, the virulence of plaque-purified 1194-SP and 1194-LP were determined in detail. 1194-SP infection resulted in 90% mice death by day eight p.i., while 1194-LP infection failed to cause any mouse death at least until day 14 p.i. (*P *< 0.001). The weak virulence of 1194-LP was also confirmed by its MLD_50 _and the mouse average survival time (Table 1). The mean survival times of both VN1194-W and 1194-SP groups were almost the same. In another experiment with a smaller dose (25 pfu) of intravenous inoculation of VN1194-W resulted in 100% of mice death, while VN1194-P and 1194-LP separately caused only 10% and 0% of mouse death. The mean survival times were significantly different between the VN1194-W group and the VN1194-P or 1194-LP group (*P *< 0.001).

### Reduced neurotropism contributed to the attenuation of VN1194-P

#### Reduced neurotropism in mice of 1194-LP or VN1194-P

**Figure 4:**
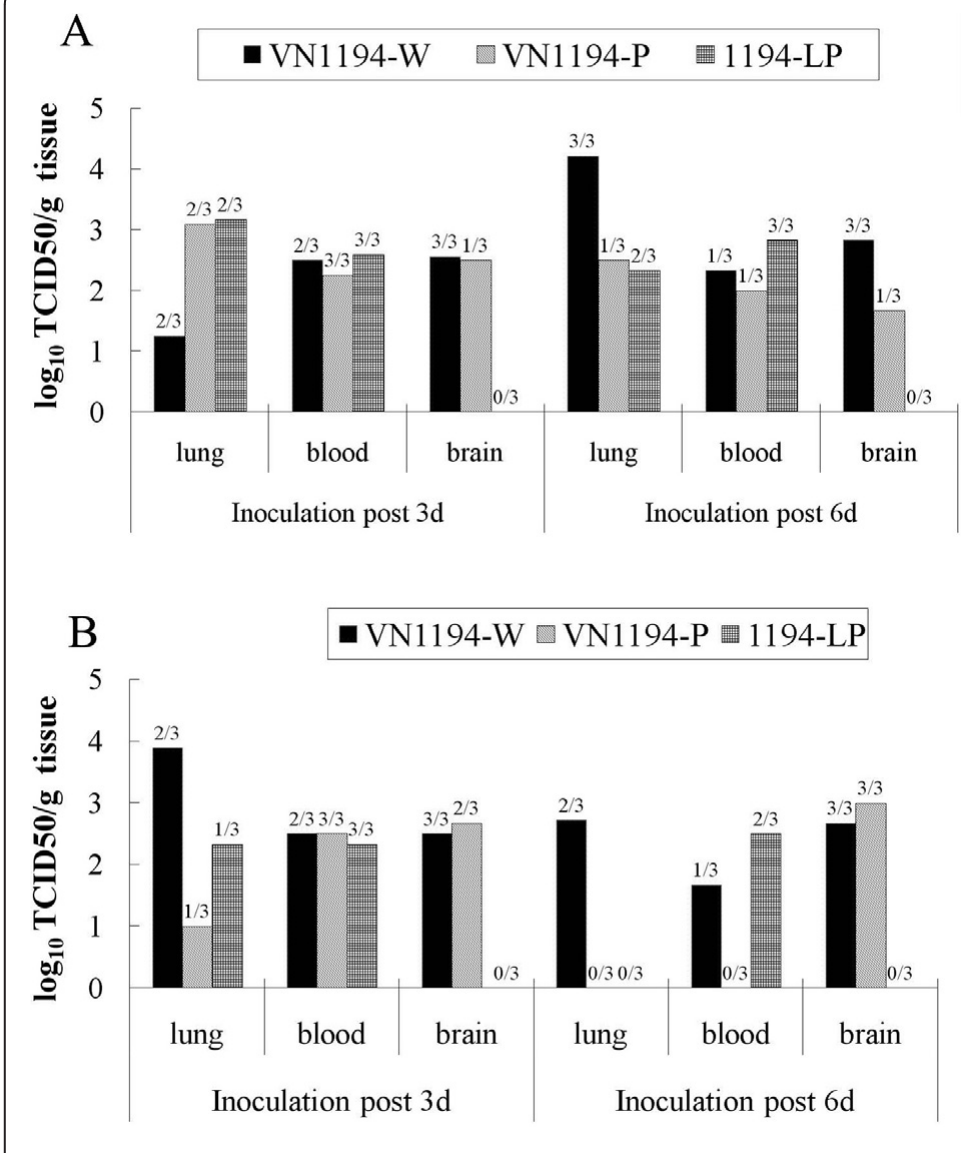
**Replication of VN1194-W, VN1194-P and 1194-LP viruses in mouse lung, blood and brain**. Mice were infected i.n. with 100 pfu (A), or i.v. with 50 pfu (B) of each virus. On days three and six p.i., three mice in each group were sacrificed and virus titers in each organs or tissues were determined by TCID_50 _on MDCK cells. The ratio of positive to total sample was labeled on the top of each histogram. A: Virus titers in the lung of mice infected with VN1194-W were significantly lower on three p.i. but significantly higher on six p.i. than those in the lung of mice infected with the other two viruses (*P *< 0.01). The differences of virus titers in mouse brain on daythree or six p.i. between the mice infected with 1194-LP and those infected with the other two viruses were also significant (*P *< 0.01). B: virus titers in the lung of mice infected with VN1194-W were significantly higher on both day three and six p.i. than those in the lung of mice infected with the other two viruses (*P *< 0.01), and the differences of virus titers in mouse brain on day three or six p.i. between the mice infected with 1194-LP and those infected with the other two viruses were also significant (*P *< 0.01).

We investigated the correlation between growth characteristics in mice and virulence in mice of those viruses (Figure 4). Because the virulence and genome sequences of 1194-SP are similar to those of VN1194-W, only VN1194-P, VN1194-W and 1194-LP were tested in following experiments. After intranasal inoculation of 100 pfu, the virus was detected on days three and six p.i. in the lungs and bloods of the mice infected with VN1194-P, VN1194-W and 1194-LP, indicating successful infection of these viruses. No virus was detected in the mice brains after 1194-LP infection, while the brains of mice infected with VN1194-P and VN1194-W were detected positive by TCID_50_. The positive rate and virus titers in mice brain of VN1194-P group were both lower than VN1194-W group. Same results got by intravenous inoculation, H5N1 virus infected mice efficiently by intravenous inoculation, and VN1194-W and VN1194-P could replicate in the lung, blood and brains, but no virus was detected by TCID_50 _in the brains of mice infected with 1194-LP.

#### High virulence of brain isolate of mice infected with VN1194-P

To further confirm whether the less neurotropism of 1194-LP isolated from VN1194-P contributed to the attenuation of VN1194-P, we separately isolated the viruses from the lung (1194-ML) and the brain (1194-MB) of mice infected with VN1194-P, and then we investigated the plaque forming property and virulence of two isolates. Similar to VN1194-P, 1194-ML also formed heterogeneous plaques, while 1194-MB only formed smaller plaques whose size was as small as those formed by VN1194-W (Figure 1E and 1F, Table 1). The virulence in mice of 1194-ML or 1194-MB was investigated with similar procedures. We found that 1194-MB was more pathogenic in mice than 1194-ML and VN1194-P (Figure 3C) (mean survival time, *P *< 0.001) but was less pathogenic than 1194-SP and VN1194-W (Table 1) (mean survival time, *P *< 0.05)

### Genetic sequences analysis

To understand the genetic basis of distinct plaque forming property and virulence of these viruses, we performed sequence analysis for the genomes of VN1194-W and other variants. As in Table 2 (fragments without amino acid differences not shown), the amino acids sequences of the ten fragments of VN1194-W and 1194-SP were all consistent with previously published sequences of A/Vietnam/1194/2004 (M1:AAT70527; M2: AAT70526; NP: AAT70629; HA: AAT73273; NA: AAT73327; NS1: AAT73394; NS2: AAT73393; PB1: AAT73495; PA: AAT74486; PB2: AAT73549) except for two amino acid substitutions, one at His^60 ^→ Asp in PB2 and another at Met^86 ^→ Ile in PA. Same substitutions were also discovered in all other variants in this study. Therefore, 1194-SP is the dominant variant of VN1194-P, inheriting all the genome sequences from VN1194-W. However, 1194-LP fragments had a few substitutions that would contribute to the virus' attenuation. We also found that both 1194-LP and 1194-ML contained three amino acid substitutions in PB2 (Asp^26 ^→ Asn, Ile^63 ^→ Thr, and Lys^189 ^→ Arg), two substitution in PB1 (Thr^677 ^→ Met, Val^709 ^→ Ile), and two substitutions in PA (Ile^30 ^→Thr and Phe^46 ^→Ser). All or part of these homologous mutations contributed to the attenuation and plaque forming property of VN1194-P and 1194-LP. In addition, 1194-LP had more substitutions than 1194-ML, including two in PB1 (Leu^143 ^→ Ser; Glu^256 ^→ Lys), two in PA (Lys^102 ^→Arg and Leu^106 ^→Pro) and four in NP (Arg^106 ^→ Ser; Ser^262 ^→ Pro; Ala^271 ^→ Thr; and Ala^366 ^→ Asp).

**Table 2:** Amino acid substitutions of VN1194-W and other viruses

Virus	PB2				PB1					PA						NP		
				
	26	60	63	189	143	256	325	677	709	30	46	86	94	102	106	262	271	366
VN1194*	D	H	I	K	L	E	I	T	V	I	F	M	I	K	L	S	A	A
VN1194-W	D	**D**	I	K	L	E	I	T	V	I	F	**I**	I	K	L	S	A	A
1194-SP	D	**D**	I	K	L	E	I	T	V	I	F	**I**	I	K	L	S	A	A
1194-LP	**N^※^**	**D**	**T**	**R**	**S**	**K**	I	**M**	**I**	**T**	**S**	**I**	I	**R**	**P**	**P**	**T**	**D**
1194-ML	**N**	**D**	**T**	**R**	L	E	I	**M**	**I**	**T**	**S**	**I**	I	K	L	S	A	A
1194-MB	D	**D**	I	K	L	E	**T**	T	V	I	F	**I**	**V**	K	L	S	A	A

*: Reference A/Vietnam/1194/2004 (H5N1) sequences downloaded from NCBI (PB2: AAT73549; PB1: AAT73495; PA: AAT74486; NP: AAT70629)

^※: ^Mutations, compared to reference sequences, were highlighted byFrame line and bold type.

## Discussion

Plaque assay is one of the most important procedures for the isolation and titration of viruses like influenza A that forms plaques on MDCK and other cell lines [27,28]. In this study, we found that VN1194-P formed heterogeneous plaques on MDCK cells, indicating a mixed population, while the less propagated VN1194-W virus formed only homogeneously small plaques. The population of larger plaque variants was initially too small in VN1194-W to be detected by plaque assay. And, with the selection by chicken embryos and (or) MDCK cells at a significantly lower temperature than that of the virus' natural bird reservoir, the population was enriched after serial proliferations, as was supported by the higher replication efficiency of 1194-LP to 1194-SP in MDCK cells. It has been previously suggested that various amino acid residues in HA1, M1, NS1 and PB1 of influenza viruses are associated with plaque formation on MDCK cells [29-33]. However, the plaque heterogeneity among viruses examined in this study did not appear to be associated with similar changes at those sites. Amino acid changes in PB2 (D26N, I63T, K189R), PB1 (T677M), and PA (I30T, F46S) were identified that may affect plaque formation in MDCK cells.

We also discovered that VN1194-W replicated in mouse brain and caused lethal infection after being introduced to the animals intranasally. And we initially observed that this virus' virulence was attenuated in mice after a few *in vitro *passages, and only a very low virus titer was determined in one of the three brains of mice after i.n. infection with the VN1194-P. Furthermore, the less virulence of 1194-LP, the larger-plaque variant from VN1194-P, was also associated with no neurotropism in mice. The isolate from the brains (1194-MB) of mice infected with VN1194-P only formed smaller plaques with high virulence in mice. Therefore, the less neurotropism of 1194-LP isolated from VN1194-P may have contributed to the attenuation of the virus.

We are interested in understanding how the virulence attenuation of 1194-LP is related to the changes in the viral proteins. Multiple residues at cleavage site of HA [34,35], the 627^th ^amino acid of PB2 [35,36], the 66^th ^amino acid of PB1-F2 [37], and the 92^nd ^amino acid of NS1 [38] have been identified as highly pathogenic markers of H5N1 viruses. However, there were several amino acid substitutions on other than above sites were found in 1194-LP and 1194-ML, both attenuated variants, including three amino acid substitutions (Asp^26 ^→ Asn, Ile^63 ^→ Thr, and Lys^189 ^→ Arg) in PB2, two substitution (Thr^677 ^→ Met, Val^709 ^→ Ile) in PB1 and two substitutions (Ile^30 ^→ Thr and Phe^46 ^→Ser) in PA, all or part of which may cause the attenuation of this virus in mice. In addition, other mutations that only happened in 1194-LP, including two in PB1, two in PA and four in NP, may also be associated with the attenuation in neurotropism in mice.

Our present study demonstrates that less virulent variants are selected by serial passages of VN1194 in embryonated eggs and MDCK cells. The virulence changes were correlated with specific amino acid changes in PB2, PB1, PA and NP proteins, which may have occurred during *in vitro *culturing. Therefore, the viruses after serial proliferations *in vitro *may not represent the original viruses in patients or other hosts, emphasizing the importance of direct analysis of original virus samples. Further study on mutations in those proteins may explain the mechanisms of the virulence or neurotropism of H5N1 influenza virus in mice.

## Competing interests

The authors declare that they have no competing interests.

## Authors' contributions

JL, BHL YH, and DWZ carried out plaque forming assay, MLD_50 _and mice survival rate and survival time of viruses. YH, YKX and YQL mainly determined the virus titers in mice tissues and virus growth curve. JL and GHC finished the genetic Sequencing of viruses. JL GHC, YHY and QYZ wrote the manuscript. All authors read and approved the final manuscript.
